# Intestinal paracoccidioidomycosis resembling Crohn’s disease in a teenager: a case report

**DOI:** 10.1186/s13256-018-1641-z

**Published:** 2018-04-30

**Authors:** Elizete Aparecida Lomazi, Leandro Minatel Vidal de Negreiros, Pedro Vitor Veiga Silva Magalhães, Raquel de Castro Siqueira Togni, Nielce Maria de Paiva, Antonio Fernando Ribeiro, Raquel Franco Leal

**Affiliations:** 1 Department of Pediatrics, University of Campinas, UNICAMP, Campinas, Sao Paulo Brazil; 2 Inflammatory Bowel Disease Research Laboratory, University of Campinas, UNICAMP, João Lopes Vieira Street, no 108, 61, Tower 2, Campinas, Sao Paulo 13087-734 Brazil

**Keywords:** Crohn’s disease, Intestinal paracoccidioidomycosis, Differential diagnosis

## Abstract

**Background:**

Differential diagnosis of inflammatory bowel disease is often very challenging. Paracoccidioidomycosis is a fungal disease that can mimic manifestations of Crohn’s disease.

**Case presentation:**

We report a case of a 13-year-old Caucasian boy with abdominal pain for 1.5 years associated with nausea, diarrhea, and weight loss of 10 kg. He presented increased C-reactive protein and an increased erythrocyte sedimentation rate. A colonoscopy showed deep serpiginous ulcers throughout his entire colon and rectum, which suggested Crohn’s disease. He received one dose of infliximab, which is an anti-tumor necrosis factor-α, and showed no improvement. After the second dose, he got worse and started to have bloody diarrhea. A new colonoscopy was performed and pathological examination revealed ulcerative chronic inflammation with non-caseating granulomas and fungal structures (budding forms) compatible with *Paracoccidioides brasiliensis.* He underwent intravenously administered and then orally administered trimethoprim-sulfamethoxazole treatment. Due to drug intolerance, he was treated with amphotericin B and itraconazole, then he showed clinical improvement and mucosal healing with good outcome.

**Conclusion:**

Paracoccidioidomycosis must be part of the differential diagnosis of inflammatory bowel diseases in endemic areas and must be excluded before starting immunosuppressive therapy.

## Background

Paracoccidioidomycosis is a fungal disease caused by *Paracoccidioides brasiliensis*, mostly restricted to Latin America [1]. It usually manifests itself more severely in children and adolescents, and has a variety of clinical manifestations, allowing for differential diagnosis with various diseases, including inflammatory bowel diseases [2–4].

The entire gastrointestinal (GI) tract can be affected by paracoccidioidomycosis, with greater impact on the small and large intestines, and endoscopic characteristics can be similar to Crohn’s disease [5–7]. Differential diagnosis is very important in endemic areas because treatments differ considerably. The patient may experience clinical worsening and even progression to death in these misdiagnosed situations.

## Case presentation

**Fig. 1:**
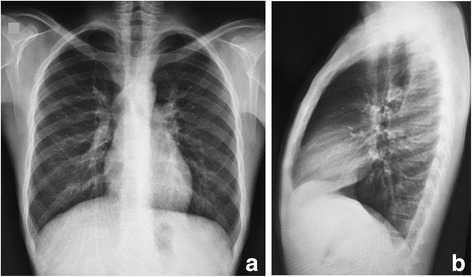
Chest X-ray imaging with no alterations. **a** Anteroposterior position. **b** Lateral position

A 13-year-old Caucasian boy, from an urban area, started follow-up in our Pediatric Gastroenterology Department with a complaint of abdominal pain of 1.5 years’ duration. He presented periumbilical pain, associated with abdominal distension, diarrhea (three to eight episodes per day), without mucus or blood, associated with nausea and a 10 kg weight loss. He had no previous history of illness or hospitalization, and he presented good psychosocial and school development. He reported no respiratory symptoms and a chest X-ray was normal (Fig. 1).

**Fig. 2:**
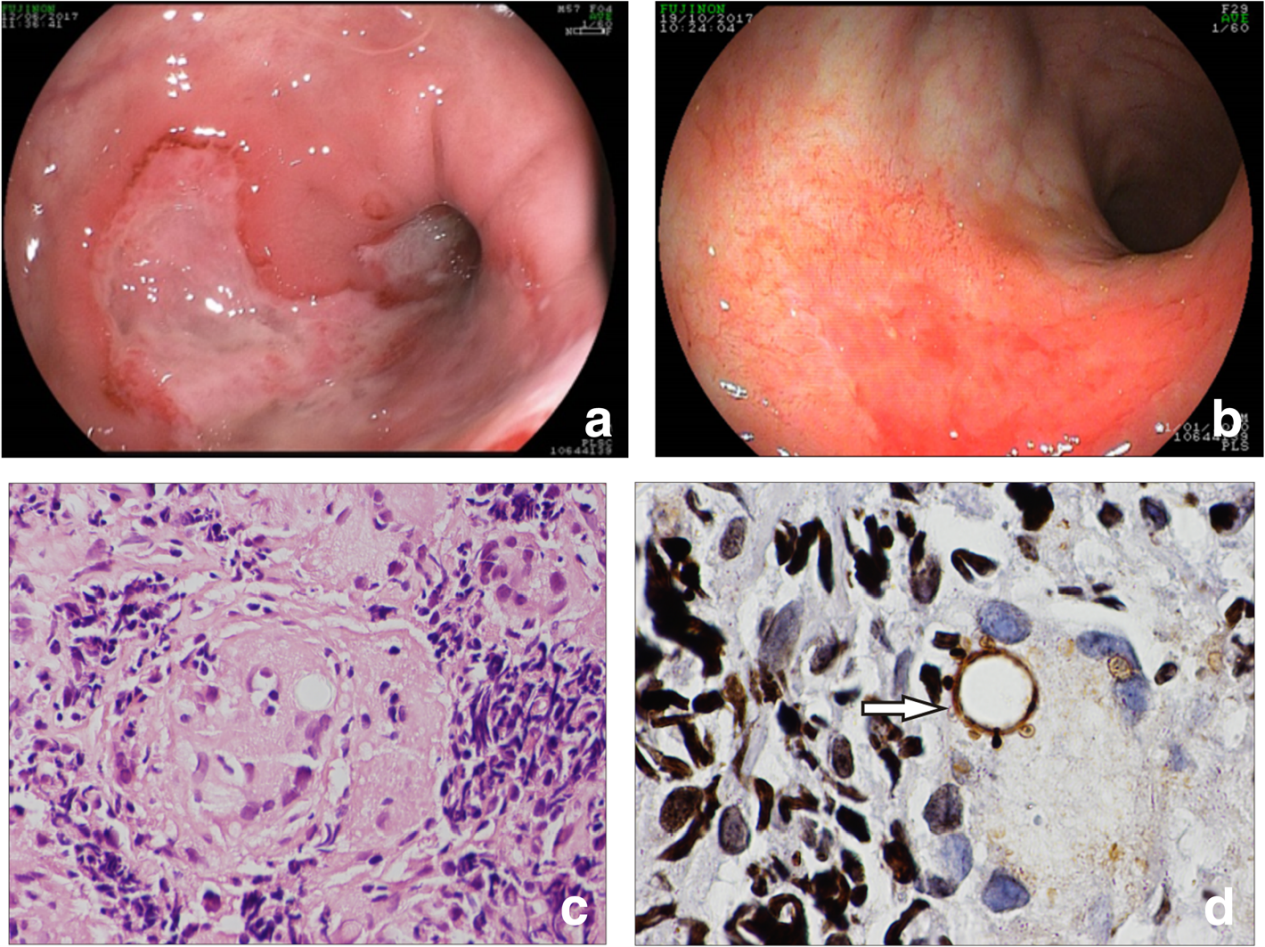
Endoscopic and histological findings of a case with paracoccidioidomycosis. **a** Colonoscopy findings at the diagnosis (large deep serpiginous ulceration). **b** Colonoscopy findings at the follow-up after antifungal therapy (mucosal healing). **c** Chronic inflammation with non-caseating granulomas, hematoxylin and eosin staining, × 400 magnification. **d** Fungal structures (budding forms) compatible with Paracoccidioides brasiliensis (arrow), Grocott staining, × 1000 magnification

At admission, he was dehydrated, swollen, and presented diffuse tenderness with abdominal palpation on physical examination. Initially, celiac disease was discarded and upper and low digestive endoscopies were requested. The upper endoscopy was normal. A colonoscopy examination showed colonic segments with multiple confluent deep serpiginous ulcerations, covered with fibrin and friability at the touch of the device, suggesting Crohn’s disease (Fig. 2a). During the colonoscopy, biopsies were performed and histopathology revealed ulcerative chronic inflammation without any other findings, despite special staining for identifying fungal forms.

An abdominal ultrasound revealed a slight hepatosplenomegaly with increased abdominal lymph nodes and a small volume of ascites, suggesting a systemic disease. The results of the laboratory tests were: hemoglobin 11.7 g/dL; hematocrit 35%; erythrocyte sedimentation rate (ESR) 53 mm/hour; C-reactive protein (CRP) 24.7 mg/L; alkaline phosphatase 63 UI/L; gamma-glutamyltransferase 9 UI/L; aspartate transaminase 12 UI/L; alanine aminotransferase 5 UI/L; urea 21 mg/dL; creatinine 0.42 mg/dL; and albumin 3.5 g/dL.

Basic infections of the GI tract were ruled out before putting our patient on biological therapy. A chest X-ray, serology for hepatitis B and hepatitis C, serology for HIV (human immunodeficiency virus), skin test for tuberculosis, fecal culture, and screening for cytomegalovirus (CMV) and *Clostridium difficile* were performed and results were all negative. Since the endoscopic findings suggested Crohn’s disease, and the first biopsies only revealed chronic inflammation without evidence of specific infection, Crohn’s disease was considered and biological therapy was started. Our patient received a first dose of infliximab, which is an anti- tumor necrosis factor (TNF)-α, and made no improvement. After the second dose, he got worse and started to have bloody diarrhea. Another colonoscopy with more biopsies was scheduled; histopathological analysis revealed ulcerative chronic inflammation with non-caseating granulomas (Fig. 2c) and with fungal structures (budding forms) compatible with *Paracoccidioides brasiliensis* (Fig. 2d). He underwent intravenously administered and then orally administered trimethoprim-sulfamethoxazole treatment, but he developed a drug reaction with eosinophilia and systemic symptoms (DRESS) syndrome, thus requiring intensive care. Due to this drug intolerance, he was treated with amphotericin B and itraconazole; he showed clinical improvement and mucosal healing as shown by a follow-up colonoscopy (Fig. 2b).

## Discussion and conclusions

Paracoccidioidomycosis is a fungal disease that can be expressed systemically, leading to several complications which may in turn lead to a variety of clinical manifestations [2]. The GI tract is affected in a minority of cases, and patients may present bloody diarrhea, abdominal pain, and malabsorption syndrome actually caused by other pathologies [5, 8]. This fungal disease is the most prevalent mycosis in Latin America [8]. These clinical manifestations depend on the patient’s immunological status [9]. Lung or intestinal involvement predominates in non-immunosuppressed patients, while widespread disease often occurs in immunosuppressed patients [10]. *Paracoccidioides brasiliensis* interacts with the host immune system and produces a complex immune response because of its antigenic structure. Activation of the complement system, natural killer (NK) cells, and other innate immunity cells confer resistance to the fungus, modulating phagocytosis [10, 11]. The production of TNF-α and interleukin (IL)-10 is associated with the activation of toll-like receptor (TLR)2 and TLR4, which recognize and internalize the fungus. The synergistic effect of these cytokines is essential to mediate fungicidal activity against the *Paracoccidioides brasiliensis* [10, 11]. TNF-α is responsible for the modulation and amplification of the immune response, promoting macrophage-mediated granulomatous reaction [12]. For this reason, the use of anti-TNF-α in a misdiagnosed patient with paracoccidioidomycosis can be very harmful. The absence of this cytokine can compromise the defense mechanisms of the formation of granulomas, which are crucial to prevent fungal multiplication [10, 12, 13].

The main differential diagnoses that should be performed are those for inflammatory bowel disease, colorectal cancer, and tuberculosis. Together, clinical manifestations and colonoscopic and histological findings can clearly simulate Crohn’s disease. Biopsy is indispensable to complete the diagnosis and to introduce specific treatment [14]. The treatment is long, the drugs may have undesirable side effects, and the first choice is sulfamethoxazole-trimethoprim [15]. In this reported case, our patient progressed with DRESS syndrome [16], requiring further hospitalization and intensive care. After sulfamethoxazole-trimethoprim discontinuation, and the introduction of itraconazole and amphotericin B, he achieved mucosal healing and disease control, showing clinical, endoscopic, and laboratorial improvement.

Our conclusion was that he had a rare disseminated paracoccidioidomycosis that affected his GI tract (his lungs were not affected), which had been misdiagnosed because clinical manifestations and colonoscopic and histological findings convincingly simulated Crohn’s disease. Even after two doses of infliximab, he did not make any improvement and actually got worse. This evolution, in addition to the prompt response to anti-fungal therapy with mucosal healing, led us to conclude that it was a primary fungal infection from the beginning. If it were a GI fungal infection in an intestinal mucosa affected by Crohn’s disease, we would not see a very fast mucosal healing with just the anti-fungal therapy.

In conclusion, paracoccidioidomycosis must become part of the differential diagnosis of inflammatory bowel diseases in endemic areas and must be excluded before starting an immunosuppressive therapy.

## Abbreviations

CMV: Cytomegalovirus
CRP: C-reactive protein
DRESS: Drug reaction with eosinophilia and systemic symptoms
ESR: Erythrocyte sedimentation rate
GI: Gastrointestinal
IL: Interleukin
NK: Natural killer
TLR: Toll-like receptor
TNF: Tumor necrosis factor

## References

[1] Martinez R. New Trends in Paracoccidioidomycosis Epidemiology. J Fungi. 2017;3(1):1. 10.3390/jof3010001 PMC5715958 29371520

[2] Ameen M, Talhari C, Talhari S. Advances in paracoccidioidomycosis. Clin Exp Dermatol. 2009;35(6):576–80. 10.1111/j.1365-2230.2009.03647.x 19874328

[3] Marques S. Paracoccidioidomycosis. Clin Dermatol. 2012;30(6):610–5. 10.1016/j.clindermatol.2012.01.006 23068148

[4] Nogueira M, Andrade G. Paracoccidioidomycosis in children and adolescents. Rev Med Minas Gerais. 2015;25(2):249–57.

[5] Penna F. Blastomycosis of the colon resembling clinically ulcerative colitis. Gut. 1979;20(10):896–9. 10.1136/gut.20.10.896 PMC1412717 533701

[6] Vieira R, Lopes A, Oliveira H, Becker Júnior O, Stanzani Júnior D, Carmo A, *et al*. Anal paracoccidioidomycosis: an unusual presentation of disseminated disease. Rev Soc Bras Med Trop. 2001;34(6):583–6. 10.1590/s0037-86822001000600014 11813067

[7] de Souza H, Fiocchi C. Immunopathogenesis of IBD: current state of the art. Nat Rev Gastroenterol Hepatol. 2015;13(1):13–27. 10.1038/nrgastro.2015.186 26627550

[8] Goldani LZ. Gastrointestinal paracoccidioidomycosis: an overview. J Clin Gastroenterol. 2011;45(2):87–91. 10.1097/MCG.0b013e3181faead4 20975575

[9] Franco M, Montenegro M, Mendes R, Marques S, Dillon N, Mota N. Paracoccidioidomycosis: a recently proposed classification of its clinical forms. Rev Soc Bras Med Trop. 1987;20(2):129–32. 10.1590/s0037-86821987000200012 3507739

[10] Benard G. An overview of the immunopathology of human paracoccidioidomycosis. Mycopathologia. 2008;165(4-5):209–21. 10.1007/s11046-007-9065-0 18777630

[11] Fortes M, Miot H, Kurokawa C, Marques M, Marques S. Imunologia da paracoccidioidomicose. A Bras Dermatol. 2011;86(3):516–24. 10.1590/s0365-05962011000300014 21738969

[12] Souto J, Figueiredo F, Furlanetto A, Pfeffer K, Rossi M, Silva J. Interferon-γ and Tumor Necrosis Factor-α Determine Resistance to *Paracoccidioides brasiliensis* Infection in Mice. Am J Pathol. 2000;156(5):1811–20. 10.1016/s0002-9440(10)65053-5 PMC1876914 10793093

[13] Parise-Fortes M, Silva M, Sugizaki M, Defaveri J, Montenegro M, Soares A, *et al*. Experimental paracoccidioidomycosis of the Syrian hamster: fungicidal activity and production of inflammatory cytokines by macrophages. Med Mycol. 2000;38(1):51–60. 10.1080/mmy.38.1.51.60 10746228

[14] Galeazzi C, Estofolete C, Moraes Filho A, Simoni A, Gonçalves-Filho F, Netinho J. Fungal Colitis by *Paracoccidioides brasiliensis*: a case report. J Coloproctol. 2011;31(4):393–6.

[15] Visbal G, San-Blas G, Murgich J, Franco H. *Paracoccidioides brasiliensis*, Paracoccidioidomycosis, and Antifungal Antibiotics. Curr Drug Targets Infect Disord. 2005;5(3):211–26. 10.2174/1568005054880118 16181141

[16] Choudhary S, McLeod M, Torchia D, Romanelli P. Drug Reaction with Eosinophilia and Systemic Symptoms (DRESS) Syndrome. J Clin Aesthet Dermatol. 2013;6(6):31–7. PMC3718748 23882307

